# Intra and interobserver reliability of the interpretation of high-resolution computed tomography on the lungs of premature infants

**DOI:** 10.1590/S1516-31802010000300005

**Published:** 2010-05-06

**Authors:** Márcia Cristina Bastos Boëchat, Rosane Reis de Mello, Maria Virgínia Peixoto Dutra, Kátia Silveira da Silva, Pedro Daltro, Edson Marchiori

**Affiliations:** I MD. Pediatric radiologist, Instituto Fernandes Figueira (IFF), Fundação Oswaldo Cruz (Fiocruz), Rio de Janeiro, Brazil.; II MD. Neonatologist, Instituto Fernandes Figueira (IFF), Fundação Oswaldo Cruz (Fiocruz), Rio de Janeiro, Brazil.; III MD. Epidemiologist, Instituto Fernandes Figueira (IFF), Fundação Oswaldo Cruz (Fiocruz), Rio de Janeiro, Brazil.; IV MD. Radiologist, Universidade Federal Fluminense (UFF), Niterói, Rio de Janeiro, Brazil.

**Keywords:** Tomography, X-ray computed, Infant, premature, Lung, Bronchopulmonary dysplasia, Reproducibility of results, Infant, Tomografia computadorizada por raios X, Prematuro, Pulmão, Displasia broncopulmonar, Reprodutibilidade dos testes, Lactente

## Abstract

**CONTEXT AND OBJECTIVE:**

High-resolution computed tomography (HRCT) of the lungs is more sensitive than radiographs for evaluating pulmonary disease, but little has been described about HRCT interpretation during the neonatal period or shortly afterwards. The aim here was to evaluate the reliability of the interpretation of HRCT among very low birth weight premature infants (VLBWPI; < 1500 g).

**DESIGN AND SETTING:**

Cross-sectional study on intra and interobserver reliability of HRCT on VLBWPI.

**METHODS:**

86 VLBWPI underwent HRCT. Two pediatric radiologists analyzed the HRCT images. The reliability was measured by the proportion of agreement, kappa coefficient (KC) and positive and negative agreement indices.

**RESULTS:**

For radiologist A, the intraobserver reliability KC was 0.79 (confidence interval, CI: 0.54-1.00) for normal/abnormal examinations; for each abnormality on CT, KC ranged from 0.05 to 1.00. For radiologist B, the intraobserver reliability KC was 0.79 (CI: 0.54-1.00) for normal/abnormal examinations; for each abnormality on CT, KC ranged from 0.37 to 0.83. The interobserver agreement was 88% for normal/abnormal examinations and KC was 0.71 (CI: 0.5- 0.93); for most abnormal findings, KC ranged from 0.51-0.67.

**CONCLUSION:**

For normal/abnormal examinations, the intra and interobserver agreements were substantial. For most of the imaging findings, the intraobserver agreement ranged from moderate to substantial. Our data demonstrate that in clinical practice, there is no reason for more than one tomographic image evaluator, provided that this person is well trained in VLBWPI HRCT interpretation. Analysis by different observers should be reserved for research and for difficult cases in clinical contexts.

## RESUMO

**CONTEXTO E OBJETIVO:**

Tomografia computadorizada de alta resolução dos pulmões (TCAR) é mais sensível que radiografias para avaliar doença pulmonar, entretanto, pouco tem sido descrito sobre a interpretação da TCAR no período neonatal ou imediatamente após. O objetivo foi avaliar a confiabilidade na interpretação da TCAR em lactentes prematuros de muito baixo peso (LPMBP, < 1500 g).

**TIPO DE ESTUDO E LOCAL:**

Estudo transversal sobre confiabilidade intra e interobservador da TCAR em LPMBP.

**MÉTODOS:**

86 LPMBP foram submetidos a TCAR. Dois radiologistas pediátricos analisaram as tomografias. A confiabilidade foi medida pela proporção de concordância, coeficiente kappa (KC) e índices de concordância positiva e negativa.

**RESULTADOS:**

Para o radiologista A, na confiabilidade intraobservador o KC foi 0,79 (intervalo de confiança, IC: 0,54-1.0) para exames normais/anormais; para cada alteração tomográfica o KC variou de 0,05 a 1. Para o radiologista B, na confiabilidade intraobservador o KC foi 0,79 (IC: 0,54-1.0) para exames normais/anormais e variou de 0,37 a 0,83 para cada alteração tomográfica. Concordância interobservador foi de 88% para exames normais/anormais e o KC foi 0,71 (IC: 0,5-0,93) e variou de 0,51-0,67 em muitos achados anormais.

**CONCLUSÃO:**

Para exames normais/anormais, as concordâncias intra e interobservador foram substanciais. Para muitos achados tomográficos, a concordância intraobservador variou de moderada a substancial. Nossos dados demonstram que, na prática clínica, não há razão para mais de um avaliador das imagens tomográficas, desde que este seja bem treinado na interpretação de TCAR de LPMBP. A análise por diferentes observadores estará reservada para pesquisa e casos difíceis no contexto clínico.

## INTRODUCTION

Radiographs are highly useful tests that health professionals can apply in hospital care for infants during the neonatal period. Interpretation of lung images frequently contributes towards the diagnosis, treatment and prognosis of neonatal and post-neonatal respiratory diseases.

Since the 1990s, advances in perinatal care have led to increased survival among extremely premature newborns. The lungs of these premature infants are in the canalicular or saccular phase of development. After birth, even when iatrogenic interventions are limited, histopathological findings indicate that the normal growth and development of immature lungs may be compromised, thereby leading to simplification of the distal areas and decreased numbers of alveoli.^1-5^

Premature newborns requiring intensive care with ventilatory assistance are at greater risk of developing pulmonary sequelae such as bronchopulmonary dysplasia (BPD),^4^ with repercussions on their future life.^6^ The etiology of BPD has not been fully established, but BPD is known to result from multiple factors including mechanical ventilation, oxygen exposure, infection, malnutrition and patent ductus arteriosus, thus leading to lung injury.^7^ Inflammation is the final common pathway for the determining factors for lung injury.^3,8^ Premature neonates also show impaired regulation of the mechanisms for repairing the injury, thus favoring the emergence of fibrosis in the affected segments.

Palta et al.^6^ correlated the clinical and radiological data obtained during hospitalization with long-term respiratory problems among very low birth weight premature infants, which they defined as the use of bronchodilators and steroids, diagnoses of asthma and rehospitalization due to respiratory problems. These authors reported that radiological findings alone were superior to clinical criteria for predicting respiratory sequelae.^6^

The lung images that have been described during the neonatal period have related mainly to chest radiographs on children with hyaline membrane disease and BPD. Studies have demonstrated that high-resolution computed tomography (HRCT) is more sensitive than radiographs for evaluating lung disease,^9,10^ but little has been described in the literature on HRCT in relation to the neonatal period or shortly afterwards, or in relation to the consistency of its interpretation.^11-17^

Studies on the reliability of chest radiographs on patients with chronic lung disease have shown wide variation in the interpretation of images by radiologists.^18-20^ Bloomfield et al. showed poor interobserver agreement for pulmonary parenchymal abnormalities on chest x-rays of premature infants in neonatal intensive care units. They concluded that for research involving radiological interpretation, the potential lack of consistency in the abnormalities analyzed should be taken into account.^18^ In 2001, Moya et al. analyzed interobserver reliability in relation to radiological BPD scores and, taking the diagnosis to be dichotomous, i.e. BPD present or absent, the kappa coefficient ranged from 0.37 to 0.63 among the observers.^20^

## OBJECTIVE

We are unaware of any previous study specifically analyzing the inter and intraobserver reliability of chest CT scan interpretations among very low birth weight infants. Our objective was to evaluate the intra and interobserver reliability of interpretations of chest CT images on very low birth weight (VLBW) premature infants, performed during hospitalization, close to the time of hospital discharge.

## MATERIAL AND METHOD

**Figure 1:**
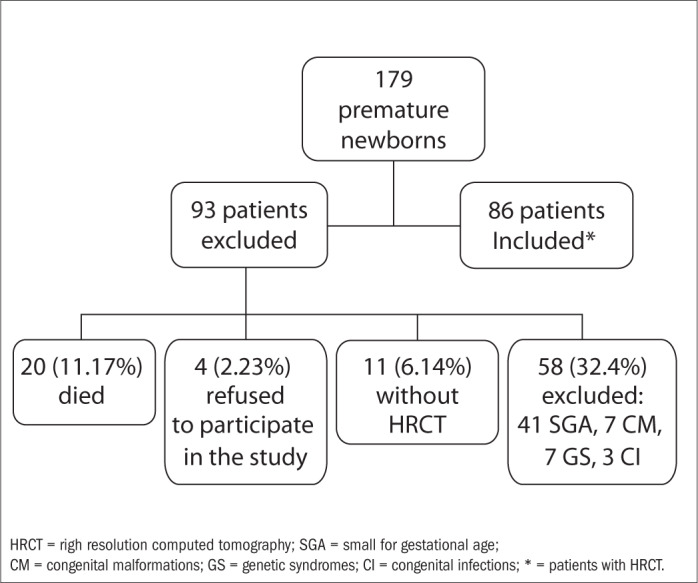
Fluxogram of patients included and excluded in this study.

This study was approved by our hospital's Research Ethics Committee. It covered HRCT performed on premature infants born between January 1, 1998, and August 31, 2000, who were admitted to a neonatal intensive care unit in a tertiary hospital in Rio de Janeiro, Brazil. During this time, 179 premature newborns were admitted to the neonatal intensive care unit. Among these, 20 (11.17%) died, and the parents of four (2.23%) refused to participate in the study. Eleven (6.14%) failed to undergo HRCT because of technical problems and 58 (32.4%) were excluded: 41 because they were small for their gestational age, seven because of congenital malformations, seven because of genetic syndromes and three because of congenital infections. This left 86 patients who underwent HRCT (Figure 1). Of these, 24 (27.9%) met the clinical diagnostic criterion for BPD, which was defined as the need for supplemental oxygen for 28 days or more.^21^ The gestational age at birth ranged from 23 to 33 weeks (mean: 28 weeks; standard deviation, SD: 2.3 weeks) and the birth weight ranged from 610-1480 g (mean: 1101 g; SD: 235 g). All the infants presented appropriate weight for gestational age, and also formed part of a prospective study to evaluate respiratory morbidity among very low birth weight premature newborns, over their first year of life.^22^

At the time of the tomographic examinations, the babies were clinically stable, breathing room air, with gestational ages corrected for prematurity that ranged from 30 to 40 weeks (mean 36 weeks; SD 2 weeks). Chest CT was performed using a ProSpeed-S™ scanner (General Electric, Milwaukee, United States), with slices of 1 mm in thickness and intervals of 10-15 mm, at 90 milliampere/second (mA/s) and 120 kilovolts (kV), without sedation and preferentially with the infant sleeping spontaneously after feeding. The technique used here was similar to the technique proposed by Seely et al. in 1997.^23^ It used more radiation than the dose recommended by Lucaya et al. in 2000^24^ because the equipment on which the examinations were performed did not allow changes to the technical parameters. Mahut et al.^16^ recently presented the results from CT scans on premature infants born between 1999 and 2001 that were performed using a similar scanner (ProSpeed Advantage™) and similar parameters to those used in our study (100 mA and a scan duration of 1.0s).

**Figure 2:**
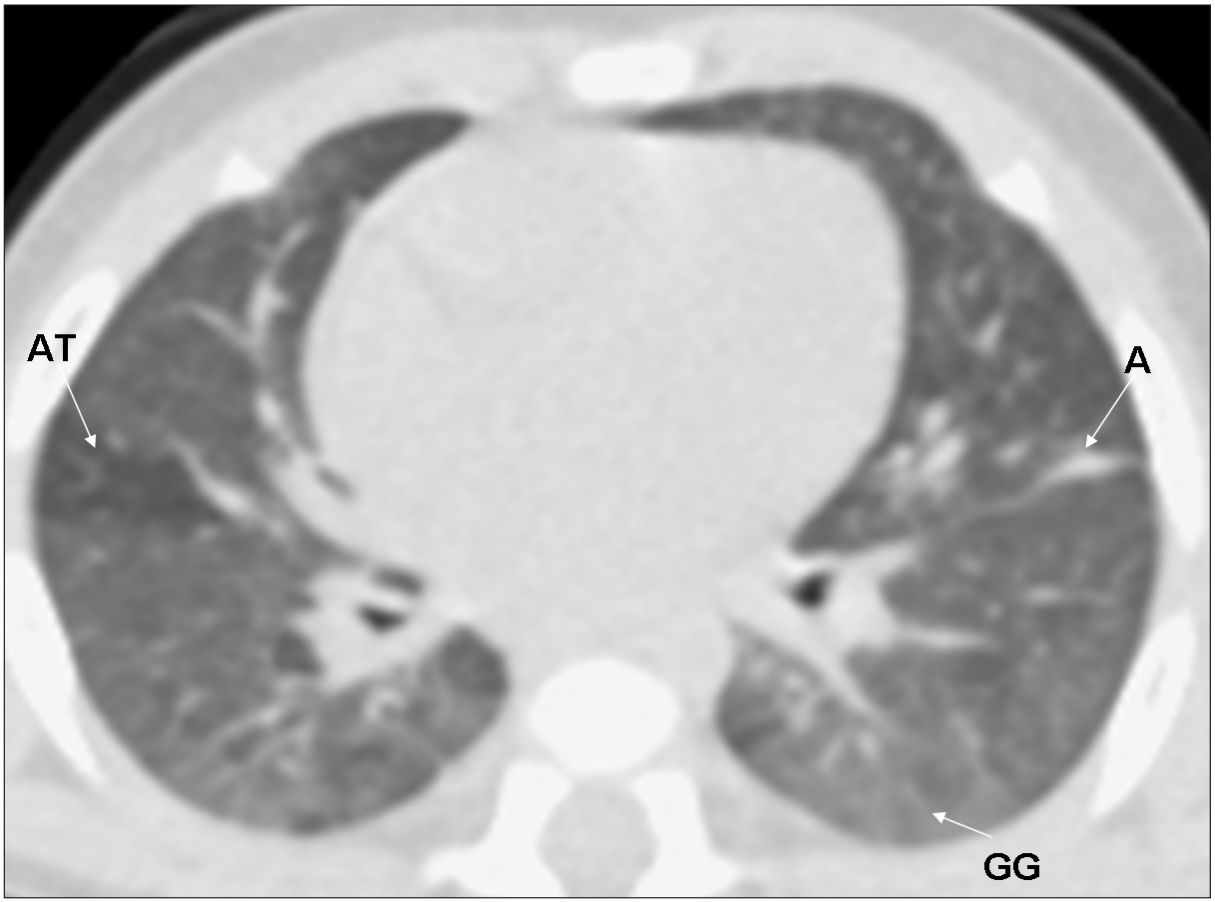
High-resolution computed tomography (HRCT) on a very low birth weight premature infant, showing air trapping (AT), atelectasis (A) and ground glass opacity (GG).

**Figure 3:**
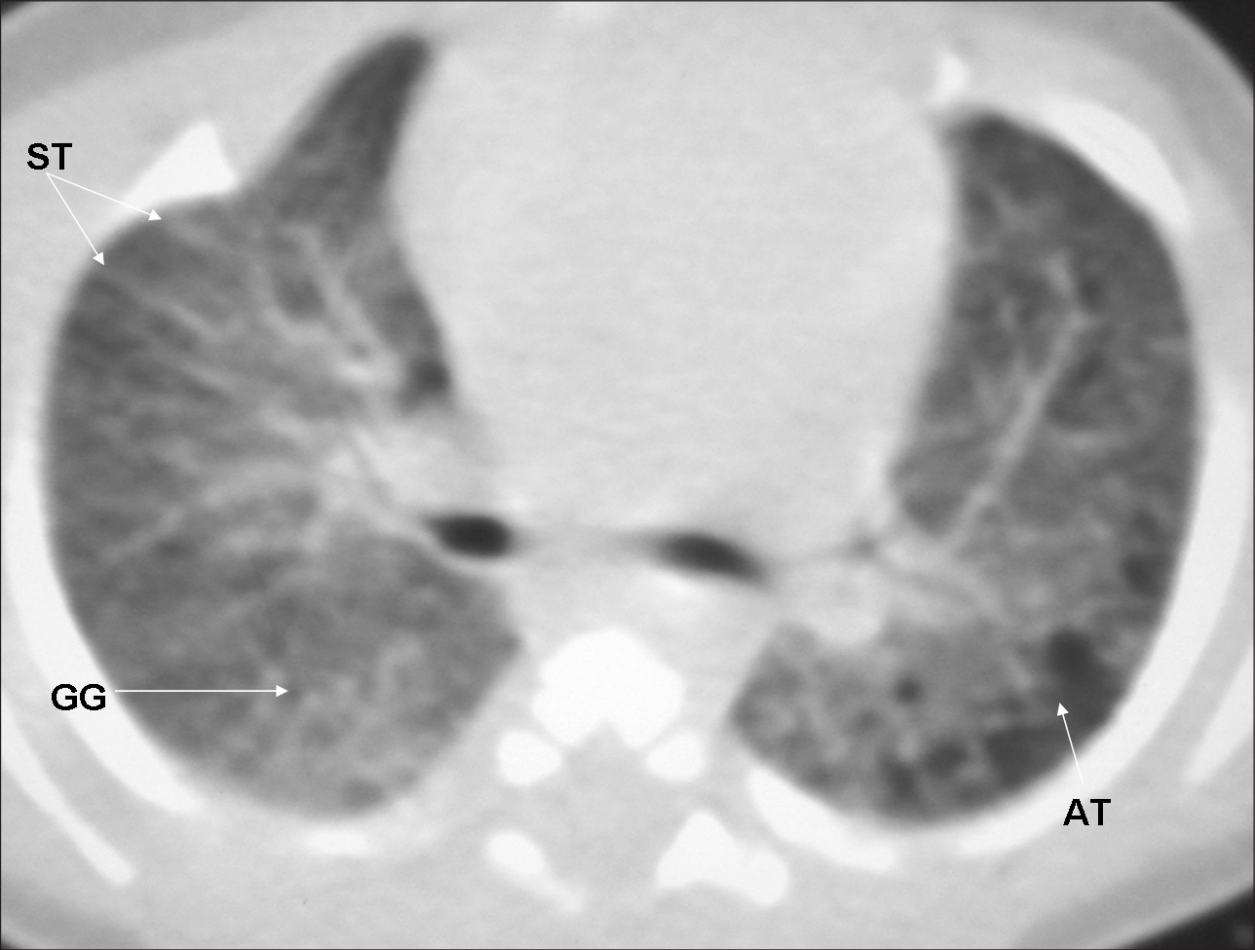
High-resolution computed tomography (HRCT) on a very low birth weight premature infant, showing air trapping (AT), ground glass opacity (GG) and septal thickening (ST).

**Figure 4:**
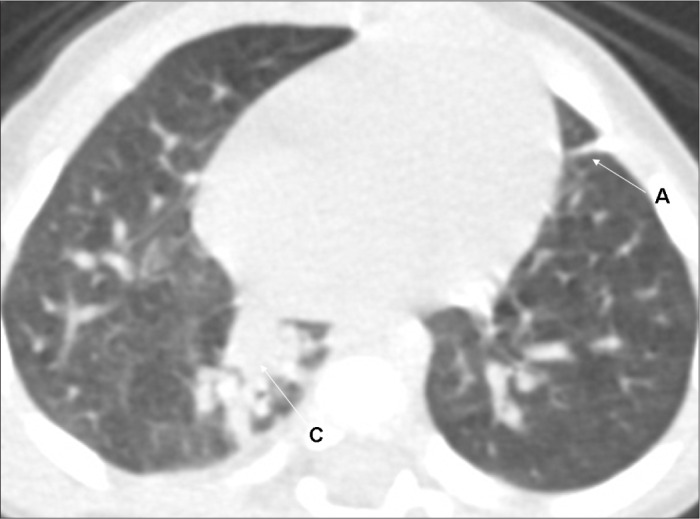
High-resolution computed tomography (HRCT) on a very low birth weight premature infant, showing atelectasis (A) and consolidation (C).

**Figure 5:**
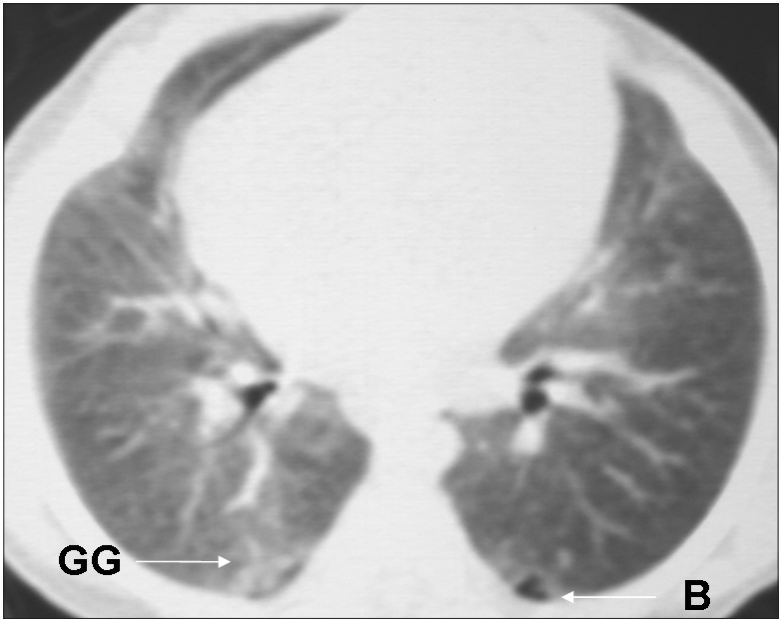
High-resolution computed tomography (HRCT) on a very low birth weight premature infant, showing bubble (B) and ground glass opacity (GG).

All the HRCT scans were read immediately after they had been produced, and radiological reports were prepared. Subsequently, for the reliability study, the films were read by two pediatric radiologists (BM, DP) with more than 10 years of experience, who were unaware of the infants’ perinatal and neonatal history. The radiologists had been previously trained, and the standardization of the abnormalities seen on CT was consistent with that reported by Webb et al.,^25^ Lucaya and Le Pointe^9^ and Oppenheim et al.,^15^ who studied the value of computerized tomography for identifying sequelae of bronchopulmonary dysplasia. The following abnormal findings were taken into account: air trapping (areas with reduced attenuation intercalated with normally attenuated areas), parenchymal band (linear opacity in the pulmonary cortex or in the corticomedullary transition of the lung, generally peripherally, with or without contact with the pleural surface), atelectasis (opacity with volumetric lung reduction due to alveolar collapse), subpleural opacity (thin curvilinear opacity, a few millimeters or less in thickness, near and parallel to the pleural surface), consolidation (increased density of the pulmonary parenchyma; most frequently homogenous and accompanied by darkening of the blood vessels), ground-glass opacity (increased density of the pulmonary parenchyma without darkening of the blood vessels), interlobular septal thickening (thin linear opaque areas between lobules, 0.1 mm in thickness) and pulmonary bubble or cyst (lesion containing air, with thin and well-defined walls; a distinction that is not always possible) (Figures 2, 3, 4 and 5).

For analysis of intra and interobserver reliability, we grouped parenchymal bands, atelectasis and subpleural opacity, because these images reflect localized volume reductions in the parenchyma. In addition, the respiratory artifacts produced during spontaneous breathing make it difficult to differentiate between these abnormalities, especially when they are small or slight.

To evaluate the intraobserver agreement, each examination was interpreted twice by the same radiologist, independently and with an interval of approximately 30 days. The radiologists did not have access to their own previous readings or those of the other radiologist. For each CT scan, the radiologists used a standard form on which they reported the presence or absence of each tomographic abnormality. The categories were not mutually exclusive, i.e. the same CT scan could show more than one abnormality.

The interobserver reliability relating to phenomena that are expressed by categorical data is frequently investigated by means of the kappa coefficient, rather than simply the joint likelihood of agreement. The degree of agreement observed between radiologists provides only an upper limit to the degree of precision in the evaluations, since the percentage of interobserver agreement can result partially from chance.^26^ Kappa values normally range from zero to one, with zero indicating agreement purely by chance and one indicating perfect agreement.^27^

Although kappa coefficients should be interpreted within the clinical context, values below zero (< 0) represent no agreement, from 0 to 0.19 poor agreement, 0.20 to 0.39 fair agreement, 0.40 to 0.59 moderate agreement, 0.60-0.79 substantial agreement and 0.80-1.00 almost perfect agreement.^28^

The inter and intraobserver reliability in reports of normal findings and the main abnormalities in CT images were measured as the proportion of agreement observed for normalcy and abnormality, the kappa coefficient, the mean positive agreement index and the mean negative agreement index. The mean positive and negative agreement indices indicate the consistency between two observers when they take positions in opposite directions regarding positive and negative decisions.^29^

## RESULTS

We analyzed HRCT scans on 86 premature infants. The clinical diagnostic criteria for BPD (defined as a need for supplemental oxygen for 28 days or more)^21^ were present in 24 children (27.9%). The mean duration of hospitalization was 59 days (median: 53 days, range 24-179 days); mechanical ventilation was used for 39 children (45.3%); and median duration of oxygen use was 149 hours.

The proportion of intraobserver agreement (radiologist A) in relation to normal and abnormal examinations was 90% and the kappa coefficient was 0.79 (confidence interval, CI: 0.54-1.0). For each abnormality seen on CT, the agreement for radiologist A ranged from 72% to 100%, and the kappa values ranged from 0.05 to 1.0. For radiologist B, the intraobserver agreement in relation to normal and abnormal readings was 92% and the kappa coefficient was 0.79 (CI: 0.54-1.0). For each abnormality seen on CT, the agreement ranged from 78% to 96% and the kappa coefficient from 0.37 to 0.83. Table 1 presents the intraobserver agreement for radiologists A and B in relation to the main abnormalities seen on CT, kappa values and confidence intervals.

**Table 1.** Intraobserver reliability in relation to abnormal chest computed tomography (CT) findings from very low birth weight premature infants

Abnormalities seen on CT	Observer A		Observer B
	Observed agreement	Kappa coefficient (CI)	Observed agreement	Kappa coefficient (CI)
Air trapping	0.76	0.51 (0.26-0.76)	0.86	0.71 (0.43-0.93)
Bands/atelectasis/ subpleural opacity	0.94	0.88 (0.63-1.13)	0.84	0.68 (0.43-0.93)
Consolidation	1	1.0 (0.73-1.27)	0.94	0.37 (0.12-0.62)
Ground glass	0.8	0.46 (0.21-0.67)	0.78	0.50 (0.23-0.77)
Septal thickening	0.72	0.05 (-0.2-0.3)	0.96	0.83 (0.58-1.08)
Bubble	0.82	0.25 (0.08-0.42)	0.94	0.78 (0.51-1.05)

CI = confidence interval.

When CT findings were classified as normal or abnormal, the proportion of agreement between the radiologists was 88% and kappa was 0.71 (CI: 0.5-0.93). Table 2 shows the reliability data between observers A and B (interobserver reliability) and the mean positive (Ppos) and negative (Pneg) agreement indices in relation to abnormal chest CT findings. The high proportion of observed agreement for interlobular septal thickening (0.69) and air trapping (0.67) was accompanied by low to moderate values for the mean positive agreement index (Ppos 0.24 and 0.55, respectively) and high values for the mean negative agreement index (Pneg 0.81 and 0.75 respectively), which tended to make the kappa correction perform worse and explains the low observed kappa values for measuring reliability for these images (0.06 and 0.34).

**Table 2.** Interobserver reliability in relation to abnormal chest computed tomography (CT) findings from very low birth weight premature infants

Abnormalities seen on CT	Observed agreement	Kappa coefficient (CI)	Ppos	Pneg	Prevalence: observer A (%)	Prevalence: observer B (%)
Air trapping	0.67	0.34 (0.16-0.52)	0.55	0.75	23.3	48.8
Bands/atelectasis/ subpleural opacity	0.83	0.67 (0.47-0.86)	0.84	0.83	51.2	53.5
Consolidation	0.91	0.54 (0.34-0.73)	0.59	0.95	7.0	12.8
Ground glass	0.78	0.53 (0.33-0.72)	0.72	0.82	38.4	39.5
Septal thickening	0.69	0.06 (-0.13-0.25)	0.24	0.81	24.4	15.1
Bubble	0.87	0.51 (0.33-0.72)	0.59	0.92	14.0	17.4

CI = confidence interval; Ppos = mean positive agreement index; Pneg = mean negative agreement index.

## DISCUSSION

The literature has shown that radiological interpretation can demonstrate variability and a certain degree of subjectivity.^18^ There is just one method or system in the medical literature for classifying pulmonary lesions by means of HRCT scans during the neonatal period.^17^ For research purposes, to reliably determine the characterization and extent of a given lesion, more than one radiologist must evaluate the lung images, in an attempt to decrease the subjectivity in their interpretation. The reliability coefficient should be used to attempt to reduce the intra and interobserver variability in imaging interpretation. When evaluating measurements with categorical responses, the kappa coefficient should be used as the agreement index.

The kappa statistic is known to compare the proportion of observed agreement in relation to the expected agreement, and depends on the prevalence of the attribute that is being measured.^27^ According to Cicchetti and Feinstein,^29^ in many situations when statistically analyzing the agreement between dichotomous data, kappa is a single index and therefore provides little information. Especially in situations with high observed agreement and low kappa (paradox), this index provides no information on the possible origins of interobserver disagreement. These authors suggested that other indicators like the mean positive and negative agreement indices should be used.^29^

The importance of discriminating between the mean positive (Ppos) and negative (Pneg) agreement indices in relation to the observed agreement is that they indicate the consistency between two observers when they take positions in opposite directions regarding positive and negative decisions. Another contribution of the Ppos and Pneg values is that they can explain the paradox of high observed agreement and low kappa through the large discrepancy between the Ppos and Pneg values. The third contribution is that when the observed agreement is high but the mean positive (Ppos) or negative (Pneg) agreement is low, correction of the kappa produces a downward adjustment, towards worse performance, thus worsening the kappa results.^29^

The intraobserver reliability in relation to normal/abnormal examinations is this study was considered substantial for both observer A and observer B (k = 0.79). For the descriptions of parenchymal bands/atelectasis/subpleural opacity (k = 0.88) and consolidation (k = 1), the intraobserver reliability (radiologist A) could be considered almost perfect. The reliability could be considered moderate for air trapping (k = 0.51) and ground glass (k = 0.46), fair for bubbles (k = 0.25) and poor in relation to septal thickening (k = 0.05). Brennan and Silman^27^ and Cicchetti and Feinstein^29^ reported that the kappa index depended on the prevalence of the attribute that was being measured. The low prevalence of these abnormalities (septal thickening and bubbles) probably contributed towards these low indices. For radiologist B, the reliability was fair just for the description of consolidation (k = 0.37). Unlike radiologist A, the intraobserver reliability index for B was substantial for air trapping (k = 0.71) and bubbles (k = 0.78), moderate for ground glass (k = 0.50), and almost perfect for septal thickening (k = 0.83).

In the intraobserver agreement for images of septal thickening, consolidation and bubbles, the kappa values were very different when comparing observer A independently from observer B. The explanation for the low kappa value in evaluating intraobserver agreement, in cases where the air trapping and interlobular septal thickening were very slight, may be that such findings were not valued by one of the observers in one of the CT scan readings.

Regarding interobserver reliability, the value (k = 0.71) found in our study for normal/abnormal examinations expressed substantial agreement between the two radiologists. For abnormal findings, we found poor agreement for interlobular septal thickening (k = 0.06) and fair agreement for air trapping (k = 0.34). However, the proportion of observed agreement for these two abnormalities (air trapping and interlobular septal thickening) was close to 70%. The kappa index is known to be affected by the prevalence of the problem in each category, in the same way as occurs in diagnostic tests (certain tests show high sensitivity and specificity, but may have low predictive accuracy when the disease prevalence is low).^30^ According to Albaum et al.,^31^ the kappa coefficient may be artificially decreased when the prevalence of the finding is very high or very low.

Perhaps the reason why there was fair agreement in air trapping but poor agreement in interlobular septal thickening was that no expiratory images were obtained, and that the examinations were performed without general anesthesia and controlled ventilation. Examinations performed without holding breath produces respiratory motion and blurred images, thereby contributing towards misinterpretation of these tomographic abnormalities. This point should be considered to be a limitation to our study.

Another possible limitation to our study was the long duration of acquisition of the CT images, which produced a higher dose of radiation and increased the secondary artifacts in the premature infants’ respiratory patterns. At the time when these scans were performed, the CT equipment did not allow changes to the technical settings, to adjust the time taken and thus the radiation dose applied to the lowest possible amounts under the circumstances. Therefore, the settings used were higher than those proposed by Lucaya et al. in 2000 (35 to 60 mA/s),^24^ but similar to those used recently by Mahut et al.^16^ with equipment equivalent to ours. The latter authors performed HRCT with settings of 100 mA and 1.0 s, on premature infants born between January 1999 and March 2001, all of which displayed criteria for BPD. Today, CT equipment only requires a very short time for image acquisition, along with low milliamperage, and this contributes towards reducing the radiation dose and produces very good images with fewer respiratory artifacts.

The age of the newborns tends to decrease the quality of some CT images, although we believe that this should not be viewed as an impediment to performing the test, which proved to be important for evaluating the extent of pulmonary involvement.

Although our study population consisted of a convenience sample, using *a posteriori* calculation of the sample size based on the CT reports (normal versus abnormal), for a null hypothesis (H_0_) with different kappa values (k = 0; k = 0.20; k = 0.40), the sample size obtained in this study was always less than the estimated *n*, and the study power (1-ß) ranged from 90% to 99%. In relation to the sample size for the kappa (k) for each type of abnormality seen on CT alteration, considering H_0_ consisting of k = 0 and 1-ß = 90%, the sample size for this study was always greater than the estimate size, with the exception of one of the CT abnormalities (septal thickening).

Few studies have presented results relating to the reliability of chest CT scans among patients who were born prematurely.^12,16,17^ It is difficult to compare our findings with the other studies because they analyzed older patients who had BPD, and used different statistical analyses. Although the contexts differed, the reliability was substantial or moderate for the majority of the abnormalities seen on CT.

Aukland et al., with technical parameters similar to those used in our study, analyzed HRCT performed in relation to apnea in two groups of patients born prematurely (10 and 18 years old) and found moderate intra and interobserver agreement (weighted kappa 0.54 and 0.52, respectively). With categorization into normal and abnormal examinations, they found substantial intraobserver agreement regarding mosaic perfusion and air trapping, and moderate to substantial interobserver agreement regarding these same tomographic findings.^12^

Ochiai et al. analyzed HRCT scans performed on premature newborns who were diagnosed with BPD.^17^ All of the tests were performed on patients with a similar age range to those in our study. To determine the interobserver reliability of the CT findings, they used kappa statistics and Spearman's rank-sum test. For mosaic patterns of lung attenuation, the kappa was poor/moderate, and for hyperaeration, it was poor/fair. For the other CT findings, the kappa ranged from fair/moderate to moderate/substantial.^17^

In the situations where we found the paradox between a high proportion of observed agreement and low kappa, other indices need to be analyzed in order to identify the sources of disagreement. Our study showed a high proportion of observed agreement and poor kappa coefficient values in relation to two types of abnormalities seen on CT: air trapping (kappa; 0.34) and interlobular septal thickening (kappa: 0.06). The values for observed agreement were close to 70%, but the mean positive agreement indices (Ppos) were 0.54 and 0.24, respectively. According to Cicchetti and Feinstein,^29^ in cases of low Ppos or Pneg values, the kappa correction adjusts downwards, towards a lower result, thereby worsening the kappa result.

## CONCLUSION

In conclusion, the intraobserver reliability in relation to normal/abnormal examinations was substantial for both observer A and observer B. The interobserver agreement was substantial for normal findings and ranged from poor to substantial for abnormal examinations (substantial for parenchymal bands/atelectasis/subpleural opacity; moderate for consolidation, ground glass opacity and bubbles; poor for interlobular septal thickening; fair for air trapping). In the case of air trapping, the mean negative agreement index (Pneg) and the mean positive agreement index (Ppos) revealed disagreement between the observers regarding the prevalence of this lesion in the study population. These values suggest that the criteria for identifying this finding need to be improved. In relation to septal thickening, for which the mean positive agreement index was 0.24 and the mean negative agreement index was 0.81, we found an even greater disagreement, thus suggesting that standardization of the criteria for defining this feature is needed.

Variability is known to be typical of radiological interpretations in the clinical context. Our data demonstrate that in clinical practice, when the observer is well trained and has experience in interpretation of HRCT on premature newborns performed without sedation and apnea, there is no reason to have more than one observer. Systematic investigation of readings by more than one radiologist would be important when image-based tests such as HRCT are used in research, or when minor components of the CT findings would make a difference in the overall diagnosis, treatment and prognosis.
